# Molecular Characterization and Epidemiology of Anaplasmataceae in Ticks and Domestic Animals in the Colombian Caribbean

**DOI:** 10.3390/ani16010008

**Published:** 2025-12-19

**Authors:** Maria Badillo-Viloria, Ignacio García-Bocanegra, Steffania de la Rosa Jaramillo, Salim Mattar, Mario Frías-Casas, David Cano-Terriza

**Affiliations:** 1 Facultad de Ciencias Básicas y Biomédicas, Centro de Investigaciones en Ciencias de la Vida, Universidad Simón Bolívar, Barranquilla 080001, Colombia; maria.badillo@unisimon.edu.co (M.B.-V.); jaramillodrs@hotmail.com (S.d.l.R.J.); 2 Grupo de Investigación en Sanidad Animal y Zoonosis (GISAZ), UIC Zoonosis y Enfermedades Emergentes ENZOEM, Departamento de Sanidad Animal, Universidad de Córdoba, 14014 Córdoba, Spain; mariofriascasas@hotmail.com (M.F.-C.); v82cated@uco.es (D.C.-T.); 3 CIBERINFEC, ISCIII CIBER de Enfermedades Infecciosas, Instituto de Salud Carlos III, 28029 Madrid, Spain; 4 Instituto de Investigaciones Biológicas del Trópico, Universidad de Córdoba, Córdoba 230002, Colombia; smattar@correo.unicordoba.edu.co; 5 Unidad de Enfermedades Infecciosas, Grupo de Virología Clínica y Zoonosis, Instituto Maimónides de Investigación Biomédica de Córdoba (IMIBIC), Hospital Reina Sofía, Universidad de Córdoba (UCO), 14004 Córdoba, Spain

**Keywords:** Anaplasmataceae, genetic diversity, tick-borne diseases, zoonoses

## Abstract

Tick-borne diseases (TBD) pose a significant threat to both animal and public health, particularly in tropical regions. In the Colombian Caribbean region, there is limited knowledge of the epidemiology of TBD in domestic animals and their vectors. In this study, conducted in northern Colombia from 2021 to 2022, we analyzed the molecular diversity of Anaplasmataceae in a total of 1156 ticks and blood samples collected from their infested hosts: 56 cattle and 17 equids (horses and mules). Polymerase chain reaction (PCR) assays were performed, using primers to amplify the mitochondrial 16S rRNA gene for tick identification and bacterial 16S and 23S rRNA to detect Anaplasmataceae. The amplified products were sequenced and analyzed for molecular characterization of species. Four tick species were identified: *Dermacentor nitens* (55.6%), *Rhipicephalus microplus* (43.0%), *Rhipicephalus sanguineus* sensu lato (0.7%), and *Amblyomma patinoi* (0.7%). Overall, 9.4% of the pooled tick samples were identified as *R. microplus*, and 64.4% of the blood samples tested positive for Anaplasmataceae. Molecular analyses identified *Anaplasma marginale* in cattle and several species in ticks, including an *Anaplasma* sp. closely related to *A. platys*-like, *Ehrlichia ruminantium*, and *E. muris* and *Ehrlichia* variants closely related to *Candidatus* E. rustica, *E. canis*, and *E. minasensis*. The results indicate high infection rates and the circulation of both well-known and potentially novel Anaplasmataceae species, suggesting complex transmission dynamics among ticks and hosts.

## Simple Summary

Tick-borne diseases represent a significant threat to both animal and public health, especially in tropical regions. Little is known about the epidemiology of these diseases in domestic animals and ticks in the Colombian Caribbean. To investigate infections caused by Anaplasmataceae bacteria, this study examined 1156 ticks and blood samples collected from 56 cattle and 17 equids in northern Colombia. Four tick species were identified, with *Dermacentor nitens* and *Rhipicephalus microplus* being the most prevalent. Around 9% of pooled tick samples and more than 60% of blood samples tested positive for Anaplasmataceae. Genetic analyses confirmed the presence of *Anaplasma marginale* in cattle and revealed several variants of *Anaplasma* and *Ehrlichia*, including *Anaplasma* sp. closely related to *A. platys*-like, *Ehrlichia ruminantium*, and *E. muris*. Some of these findings represent potentially novel variants in Colombia and highlight the complexity of transmission cycles between ticks and their animal hosts.

## 1. Introduction

Ticks and tick-borne diseases (TBD) have a considerable impact on both livestock and public health, especially in tropical regions [1,2]. These diseases significantly reduce livestock productivity, leading to economic losses for farmers and increased veterinary costs [3,4,5]. At the same time, frequent human–animal interactions mean that TBDs are also a major public health concern [2]. Changes in the dynamics at the human–animal–vector–environment interface facilitate the spread of ticks and tick-borne pathogens (TBP) [6,7]. Agricultural expansion and intensification, land-use changes, population density variations, water and food availability, outdoor human activities, and habitat fragmentation are some of the factors likely to influence the prevalence and distribution of TBDs in a world undergoing global change [6,8,9].

The main bacterial species transmitted by ticks belong to the orders Spirochaetales and Rickettsiales [10,11,12,13]. Within the order Rickettsiales, the genera *Anaplasma* and *Ehrlichia* (family: Anaplasmataceae) comprise eight and six well-recognized species, respectively, as well as recently proposed candidate species, and unclassified genovariants that infect ticks, domestic animals, wildlife, and humans [14,15].

*Anaplasma* spp. and *Ehrlichia* spp. have been detected in different animal species and humans throughout Latin America [16,17,18,19], posing significant challenges to animal and public health in endemic regions [20]. Among these pathogens, *Anaplasma phagocytophilum* and *Ehrlichia chaffeensis* are of particular concern to public health [11,15,21]; *Anaplasma marginale* is mainly associated with the livestock industry, since its high prevalence and role as the primary etiological agent of bovine anaplasmosis are responsible for considerable economic and production losses [22,23,24].

Colombia is an ideal environment for ticks and tick-borne diseases, due to its vast territory, ecological diversity, and favorable climatic conditions [25,26,27,28,29]. Molecular research on domestic animals to date has predominantly focused on dogs [2,24,30,31,32] but there are very few studies on other domestic animal species in Colombia. A comprehensive understanding of the interactions between pathogens, hosts, and vectors involved in transmission cycles is essential for elucidating the eco-epidemiology of Anaplasmataceae and developing effective control strategies. This is hindered by our current state of knowledge.

The aim of the present study was to characterize the molecular and epidemiological features of Anaplasmataceae species infection in domestic animals and ticks in the Colombian Caribbean region of northern Colombia.

## 2. Materials and Methods

### 2.1. Study Design, Sampling, and Specimen Collection

From 2021 to 2022, a cross-sectional study was conducted on mixed herds of cattle and equids in eight municipalities representative of the five subregions and main livestock-producing areas of the Department of Atlántico in the Colombian Caribbean region of northern Colombia. Livestock production in the Department of Atlántico is dominated by small, low-input, dual-purpose herds, and most farms maintain fewer than 50 productive and working animals [33,34]. All the farms primarily raised cattle while also keeping working equids (horses and mules) for herding and transportation. The study region has a tropical dry to sub-humid climate (mean annual temperature ~27 °C; annual rainfall 500–1500 mm). There is a marked bimodal rainfall pattern in May and September–November, as well as prolonged dry seasons [35].

Each farm was visited only after first obtaining the owner’s consent. Whole-herd inspections were attempted, but animal handling restrictions often limited access. Consequently, sampling was performed using a convenience-based approach, based on the specific conditions of the farm and the feasibility of handling the animals. The approach prioritized cattle in production, such as lactating cows, and resident working equids. In total, 142 host animals were examined: 116 cattle, 23 horses, and 3 mules, representing a median of 14.2 hosts per farm (range 6–28). To assess tick infestation, each animal was inspected systematically for approximately 5–10 min. The presence or absence of ticks was recorded, and specimens were carefully removed using sterile forceps, regardless of developmental stage or engorgement level. Standard half-body collection methods from predilection sites (legs, abdomen, tail, anal area, neck, lateral and dorsal regions, and ears) were used [36].

The ticks were collected in sterile tubes labeled according to location and host, preserved at 4 °C, and then transported to the laboratory for identification using morphological identification keys [37,38,39]. The specimens were grouped into pools according to species, developmental stage, host, and collection site. Each pool contained 1–12 adult ticks or 10–30 immature ticks (nymphs or larvae), depending on the size of the tick and degree of engorgement.

In addition, blood samples were drawn from 56 cattle, 14 horses, and 3 mules via coccygeal or jugular venipuncture. The samples were collected in EDTA tubes, transported to the laboratory under refrigeration conditions, and stored at −20 °C until analysis. Individual data on host species, sex, and age were collected. Herd-level epidemiological information was also collected, including location, management system, tick control measures, grazing rotation, contact with wild species, and type of feed.

### 2.2. Molecular Identification of Ticks and Molecular Analysis of Anaplasmataceae

To confirm the morphological identification of tick species, six tick pools were randomly selected based on their geographical location and analyzed using PCR and partial sequencing of the 16S rRNA gene, as previously described [40,41].

A previous screening PCR was performed on ticks and blood samples using primers and protocols previously established to detect a 345 bp fragment of the 16S rRNA gene of Anaplasmataceae [40,42]. PCR analysis revealed the presence of Anaplasmataceae DNA in the samples, which were then analyzed using PCR protocols targeting a 649 bp fragment of the 23S rRNA gene of Anaplasmataceae [40,43]. For each PCR, 5 μL of molecular-grade water was used as a negative control, and DNA from *A. phagocytophilum*, previously identified at the Instituto de Investigaciones Biológicas del Trópico (IIBT, Montería, Colombia), was used as a positive control for Anaplasmataceae.

### 2.3. Sequencing, Phylogenetic Analysis, and Genetic Divergence

Nine PCR 23S rRNA-positive DNA products were selected and sequenced: six were from pools consisting exclusively of adult ticks (1–10 individuals per pool) and three were from host blood samples. Selection of these samples was based on co-detection criteria in both the host and the infesting ticks, giving priority to cases in which both the host’s blood and the corresponding tick pools tested positive for Anaplasmataceae. This strategy was intended to improve the interpretation of host–vector relationships. All PCR products were purified using ExoSAP (Thermo Fisher Scientific, Waltham, MA, USA) according to the manufacturer’s instructions and then sequenced using the Sanger method at an external laboratory. The 23S rRNA sequences obtained were edited and assembled using MEGA X (version 11) software [44]. The consensus sequences were compared with sequences available in the GenBank database using BLAST (http://www.ncbi.nlm.nih.gov/BLAST/; accessed on 16 October 2025), and unique partial sequences were deposited in GenBank using the BankIt v3.0 NCBI submission tool. Alignments were constructed using the MAFFT tool in UGENE 53.0 software and default automatic settings [45]. Available sequences for *Anaplasma* spp. and *Ehrlichia* spp. from other geographic regions were incorporated and then manually edited.

Phylogenetic analysis was conducted using IQ-TREE2 (v2.2.2.6). The ModelFinder tool in IQ-TREE was used for automatic model selection. Maximum-likelihood (ML) analysis was performed with IQ-TREE2, and branch support was assessed using the ultrafast bootstrap (UFBoot) approximation (1000 replicates). The best-fit substitution model for each partition was determined using ModelFinder [46], based on the Bayesian Information Criterion (BIC). Each gene was partitioned individually according to the best-fit model: Ticks 16S rRNA: TPM2u+F+G4; *Anaplasma* 23S rRNA: TIM3+F+G4; *Ehrlichia* 23S rRNA: HKY+F+R2. The resulting phylogenetic trees were visualized using iTOL (v5) and annotated using Affinity.

A pairwise distance matrix (*p*-distance) was created using Mega X software to estimate the distance between sequences detected in this study and those from other Anaplasmataceae species included in the phylogenetic trees [44]. The resulting pairwise *p*-distance values were then compared to assess the genetic divergence between the sequences obtained in this study and related sequences from GenBank.

### 2.4. Statistical Analysis

The frequency distribution of tick species and their respective hosts was determined. Prevalence for ticks was calculated as the proportion of Anaplasmataceae-positive pools out of the total number of pools tested; prevalence for animals was the proportion of positive hosts out of the total number of individuals examined, with 95% confidence intervals (95% CI). The percentage of infested animals per farm was calculated as the proportion of animals carrying ticks out of the total number of animals examined on each farm.

Pearson’s chi-squared test or Fisher’s exact test (for variables with fewer than six observations in any category) was used to examine variations in prevalence relative to explanatory variables. Continuous variables were categorized according to the 33rd and 66th percentiles. All explanatory variables underwent an initial screening, and those with a *p*-value < 0.10 were retained for multivariate analysis. Collinearity between pairs of variables was assessed using Cramer’s V coefficient. The selected variables were entered into a multiple logistic regression model to identify potential risk factors for Anaplasmataceae exposure, and the model was re-run until all the remaining variables reached statistical significance (*p*  <  0.05). Model fit was confirmed using the Hosmer–Lemeshow goodness-of-fit test [47]. Statistical analyses were performed using SPSS 28.0 software (Statistical Package for Social Sciences, Inc., Chicago, IL, USA) and GraphPad Prism 8 software (San Diego, CA, USA).

## 3. Results

### 3.1. Identification and Molecular Characterization of Ticks

Ticks and blood samples were collected from a subset of 73 tick-infested hosts, with a median of 7.3 hosts per farm (range: 4–16). A total of 1156 ticks, grouped into 159 pools, were collected and identified morphologically. The mean number of tick pools was 15.9 ± 7.2 per farm (95%CI: 10.7–21.1; range 7–27) and 2.3 ± 1.5 per animal host (95%CI: 1.9–2.6; range: 1–7). The mean number of ticks per farm was found to be 115.0 ± 61.4 (95%CI: 71.7–159.5; range 26–207), while the mean number of ticks per animal host was 16.5 ± 16.9 (95%CI: 12.5–20.6; range: 2–75).

Table 1 shows the distribution of tick species by sex and developmental stage, as well as their respective host species. Four tick species were identified: *Dermacentor nitens* was the most prevalent species (55.6%), primarily collected from horses and mules, followed by *Rhipicephalus microplus* (43.0%), mainly collected from cattle. *Rhipicephalus sanguineus* sensu lato and *Amblyomma patinoi* were also found on cattle, with each accounting for 0.7% of the total specimens.

**Figure 1:**
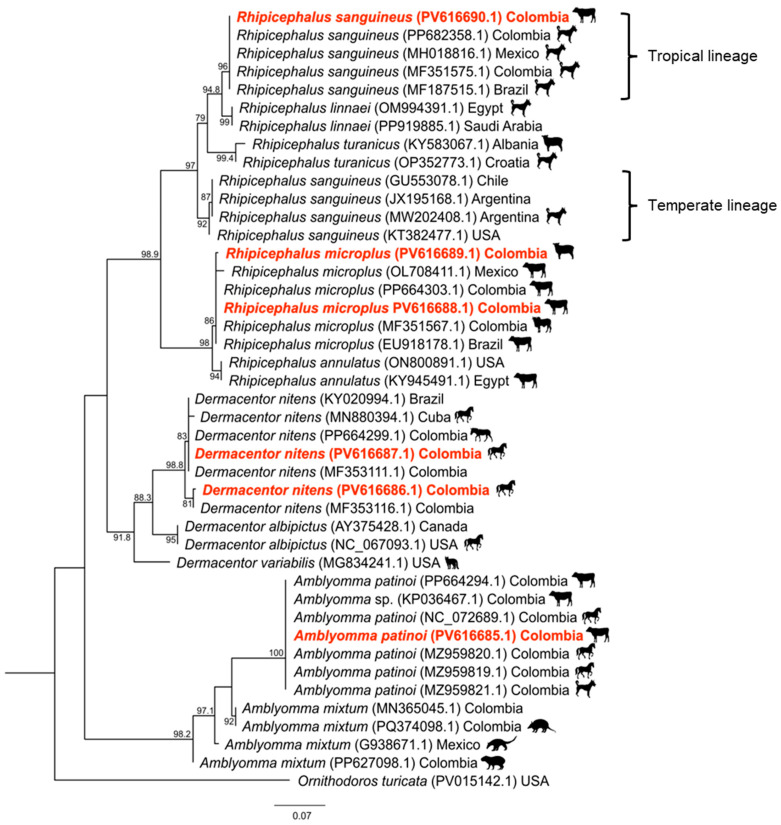
Maximum-likelihood phylogenetic tree (ML: 1000 bootstrap replicates) inferred from the partial 16S rRNA genes of ticks. Sequences identified in this study are shown in bold red.

Tick species were confirmed both morphologically and molecularly by partial sequencing of the 16S rRNA gene (Figure 1). The *R. sanguineus* s.l. sequence (PV616690.1) showed 99.5–100% identity with previously reported tropical lineage sequences from *R. sanguineus* collected from dogs in Colombia (PP682358.1, MF351575.1), Mexico (MH018816.1), and Brazil (MF187515.1). By contrast, similarity with temperate lineage sequences from Chile (GU553078.1), Argentina (MW202408.1, JX195168.1), and the USA (KT382477.1) was found to range from 93.6% to 94.2%. Comparisons with other closely related species, including *Rhipicephalus linnaei* (97.8%), *R. turanicus* (93.2–93.8%), and *R. annulatus* (87.7%), showed lower levels of similarity.

The *R. microplus* sequences (PV616688.1 and PV616689.1) exhibited 99.7–100% identity with other *R. microplus* sequences found in cattle in the Colombian region of Arauca (MF351567.1) and elsewhere in Atlántico (PP664303.1), as well as other American countries such as Mexico (OL708411.1) and Brazil (EU918178.1). Lower similarity values were observed with *R. annulatus* (97.3–97.8%) and more distantly related species, such as *R. linnaei* and *R. turanicus* (87.6–88.3%) (Figure 1).

The sequences of *D. nitens* (PV616686.1; PV616687.1) were 99.2–100% identical to those of *D. nitens* isolates found in Colombia (PP664299.1, MF353111.1, MF353116.1), Brazil (KY020994.1) and Cuba (MN880394.1). They showed lower levels of similarity with *D. albipictus* (92.5–93.9%) and *D. variabilis* (91.8–92.3%) (Figure 1).

Finally, the *A. patinoi* sequence (PV616685.1) was found to be 100% identical to previously reported *A. patinoi* and *Amblyomma* sp. sequences associated with cattle and horses in Colombia (MZ959819.1, MZ959820.1, NC072689.1, PP664294.1, KP036467.1). In contrast, *Amblyomma mixtum* (92.7–93.0%) showed lower similarity (Figure 1).

### 3.2. Anaplasmataceae in Ticks and Hosts

**Figure 2:**
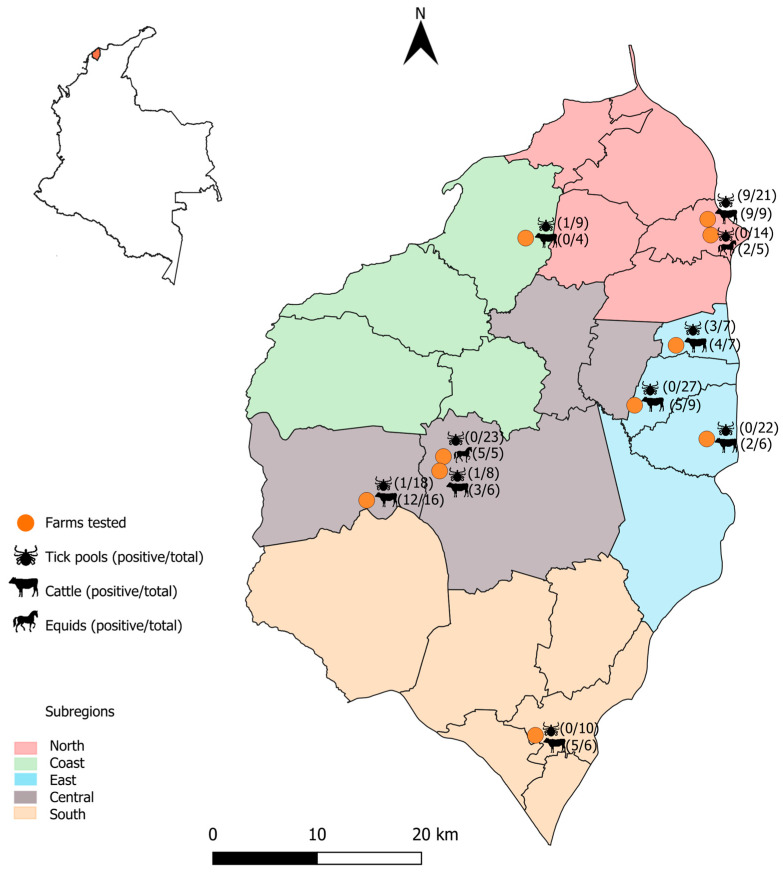
Geographic distribution of sampled farms and infection rates of Anaplasmataceae across host and tick species in the Department of Atlántico, Colombia.

Of the 159 tick pools collected, 15 (9.4%; 95%CI: 5.0–14.0) tested positive for Anaplasmataceae by 16S rRNA PCR; all of these corresponded to *R. microplus* ticks collected from cattle (18.3%; 95%CI: 10.0–27.0) (Table 1). Forty-seven of the 73 blood samples taken from the infested tick hosts (64.4%; 95%CI: 53.0–76.0) tested positive for Anaplasmataceae by 16S rRNA PCR; of these, 40 were from cattle and seven from horses (Table 1). Figure 2 shows the geographic distribution of the sampled farms and infection rates in different host and tick species. Of the 47 positive hosts, 19.1% (9/47) had at least one positive tick pool, whereas 80.9% (38/47) were only associated with negative pools. Conversely, 7.7% (2/26) of negative hosts were associated with positive tick pools, and 92.3% (24/26) exclusively with negative pools.

Table 2 shows the variables associated with Anaplasmataceae infection in the sampled animals. Four explanatory variables were selected for multivariate analysis (*p* < 0.10): “host species”, “location”, “percentage of infested animals on the farm”, and “grazing rotation”. The final multiple logistic regression model identified “host species” and “percentage of infested animals on the farm” as the main risk factors potentially associated with Anaplasmataceae infection (Table 3). The prevalence of infection was significantly higher in cattle (71.4%) than in horses (50.0%) (*p* = 0.008). Furthermore, animals on farms where ≥78% of animals were tick-infested had a 9.1-fold higher risk of Anaplasmataceae infection (80%; *p* = 0.002) than those on farms with lower infestation rates (Table 2 and Table 3).

### 3.3. Identity, Phylogenetic, and Genetic Divergence of Anaplasmataceae

A total of nine Anaplasmataceae 23S rRNA sequences were obtained. Four of these were related to *Anaplasma* spp., including one from *R. microplus* (PV595969) and three from cattle (PV595970, PV595971, and PV595972). The remaining five sequences from *R. microplus* ticks were related to *Ehrlichia* spp. (PV595967, PV595968, PV595973, PV595974, and PV595975). Interestingly, none of the Anaplasmataceae species detected on cattle matched those identified in ticks collected from the same animals.

**Figure 3:**
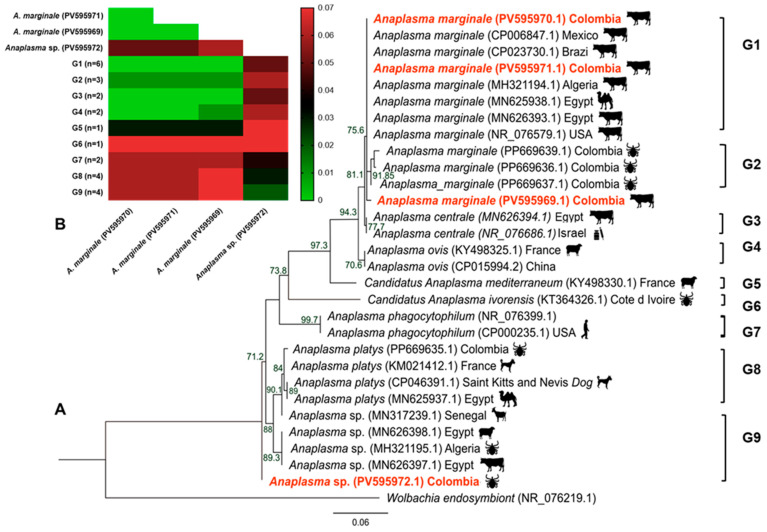
Phylogenetic tree and *p*-distances for *Anaplasma* spp. (**A**) Maximum-likelihood phylogenetic tree based on the partial 23S rRNA gene of *Anaplasma* spp. (**B**) Heatmap of mean *p*-distances. The respective values for each pairwise comparison are shown in each cell. Isolates from this study are highlighted in red. The color scale represents genetic distance: green shades indicate lower distances (higher similarity); red shades indicate higher distances (lower similarity). Abbreviations: *A.: Anaplasma;* G: Group of sequences obtained from GenBank; n: number of samples.

The first sequence, identified as *Anaplasma* sp., was isolated from *R. microplus* and formed a separate clade with *Anaplasma* sp. isolates closely related to *Anaplasma platys*-like bacteria (98.73% identity; bootstrap 92%), which have previously been detected in *Rhipicephalus annulatus* from Algeria (MH321195.1), and in cattle and sheep from Egypt (MN626398.1; MN626397.1). This clade was also closely related to the *A. platys* clade isolated previously from *R. sanguineus* in Colombia (PP669665.1) (96.84–97.71% identity; bootstrap 84%) (Figure 3A). Genetic divergence within these clades ranged from 0.02 to 0.04 (Figure 3B).

The phylogenetic tree showed that the three remaining *Anaplasma* sequences from cattle belonged to a major clade alongside *A. marginale* isolates from Africa and the Americas. This clade was further divided into two subclades. One of these exhibited 99.59–100% identity (bootstrap 86%) with *A. marginale* strains detected in cattle from the USA (NR_076579.1), Mexico (CP006847.1), and Brazil (CP023730.1), and in cattle and camels from Algeria and Egypt (MH321194.1, MN625938, MN626393.1). The other subclade included sequences previously isolated in *R. microplus* in Colombia and showed 98.30–99.33% identity (bootstrap 98%) (Figure 3A). Genetic divergence within these clades ranged from 0.00 to 0.03 (Figure 3B). The minimum and maximum divergence rates between the four *Anaplasma* spp. sequences in this study were 0.00 and 0.06, respectively (Figure 3B).

**Figure 4:**
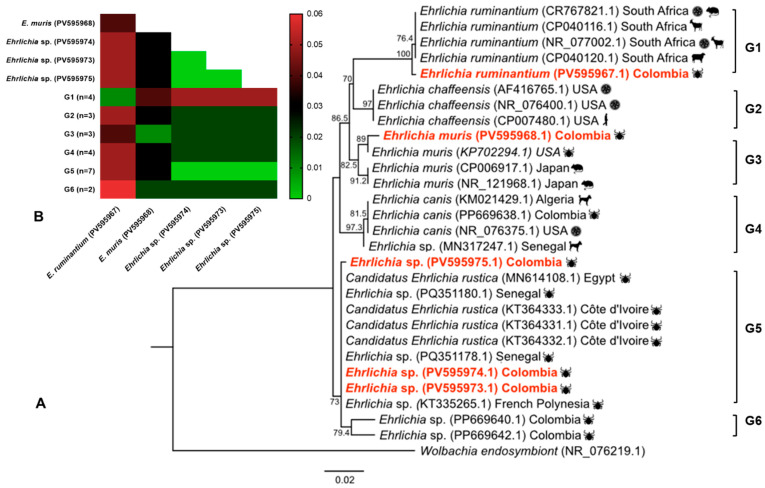
Phylogenetic tree and *p*-distances for *Ehrlichia* spp. (**A**) Maximum-likelihood phylogenetic tree based on the partial 23S rRNA gene of *Ehrlichia* spp. (**B**) Heatmap of mean *p*-distances. The respective values for each pairwise comparison are shown in each cell. Isolates from this study are highlighted in red. The color scale represents genetic distance: green shades indicate lower distances (higher similarity); red shades indicate higher distances (lower similarity). Abbreviations: *E.: Ehrlichia;* G: Group of sequences obtained from GenBank; n: number of samples.

For the *Ehrlichia* spp. sequences, phylogenetic analysis revealed that one sequence was placed within a clade containing *Ehrlichia ruminantium* isolates previously reported in *Amblyomma hebraeum* on sheep and goats from South Africa (CR767821.1, CP040120.1, CP040116.1, NR_077002.1) and showed 99.70% identity (bootstrap 100%) (Figure 4A). The genetic distance within this clade was 0.01 (Figure 4B).

Another sequence of *Ehrlichia* was clustered within a major clade containing *Ehrlichia muris* isolates (bootstrap 82%). The clade was further divided into two subclades, one including *E. muris* sequences previously reported in *Ixodes scapularis* from the USA (KP702294.1), with 99.23% identity, while the other comprised a sequence obtained from a wild mouse (*Eothenomys kageus*) from Japan (CP006917.1, NR121968.1), with 97.84% identity. The genetic distance within this clade was 0.01 (Figure 4A). The remaining three sequences, identified as *Ehrlichia* sp., formed a major clade that was divided into two subclades. One subclade included isolates of *Candidatus* Ehrlichia rustica and *Ehrlichia* sp., which were genetically close to *Ehrlichia canis*, *E. minasensis* and *E. chaffeensis*, previously reported in *Hyalomma truncatum* from Senegal (PQ351178.1, PQ351180.1), *R. annulatus* from French Polynesia (KT335265.1), *R. microplus* and *H. truncatum* from Côte d’Ivoire (KT364331.1, KT364332.1, KT364333.1), and *R. microplus* from Egypt (MN614108.1) (98.68–100% identity; bootstrap 73%).

The other subclade showed 97.00–97.65% sequence identity with *Ehrlichia* sp., which has previously been reported in *R. microplus* in Colombia (Figure 4A). Genetic divergence within these clades ranged from 0.00 to 0.02 (Figure 4B). The minimum and maximum genetic divergence values among the five *Ehrlichia* spp. sequences analyzed were 0.00 and 0.05, respectively (Figure 3B). None of the seven samples of horse blood that tested positive for Anaplasmataceae DNA yielded interpretable sequencing results.

## 4. Discussion

To the authors’ knowledge, this is the first study conducted in the Caribbean area of Colombia to investigate the community of Anaplasmataceae bacteria in competent vectors and animal hosts simultaneously. This integrative approach provides novel molecular and epidemiological insights into pathogen–host–vector interactions in the Colombian Caribbean that are important for both human and animal health, particularly in the context of livestock production.

Our results showed a markedly lower prevalence of Anaplasmataceae in tick pools (9.4%) than in host animals (64.4%) (*p* < 0.001). There was limited concordance between host and tick infection status, with only 19.1% (9/47) of positive animals having at least one positive tick pool. Despite the relatively small sample size, our findings are consistent with previous reports indicating that pathogen detection in ticks often underestimates host infection status. Factors that account for this include the unequal distribution of tick burden among individual hosts and the use of alternative transmission routes involving other arthropods such as flies and dipterans [48,49].

Some ticks may have acquired the pathogen during earlier life stages, such as the larval or nymphal stage, when blood intake is minimal, making detection less likely. In other cases, the pathogen may have been present but remained undetected due to the degradation of residual DNA during digestion [50]. Together, these findings underscore the fact that pathogen detection in ticks and hosts may not coincide in time, which limits the sensitivity of single-timepoint sampling under field conditions.

All Anaplasmataceae-positive ticks were identified as *R. microplus* (18.3%), the most prevalent cattle-infesting tick species in Colombia [51]. These findings are consistent with previous reports of up to 40% Anaplasmataceae prevalence in *Rhipicephalus* spp. [25,26,40]. *R. microplus* is recognized as a competent vector and reservoir of some Anaplasmataceae species, such as *A. marginale*, with documented evidence of transovarial transmission and co-infection events [52,53]. By contrast, *D. nitens*, the most prevalent tick species obtained in our study, tested negative for Anaplasmataceae or showed a very low prevalence, which is consistent with previous reports in Colombia [23,28,29]. This suggests that, despite its high abundance, *D. nitens* probably plays a limited role in transmitting these agents.

The frequency of Anaplasmataceae infection detected in cattle was high (71.4%). Previous studies carried out in other Caribbean regions of Colombia using microscopy and/or molecular methods have reported lower prevalence rates in cattle, ranging from 20.6% to 59.0% [23,24,49,54,55,56,57]. Future studies involving a greater number of animals are needed to more accurately assess the situation in the study region.

None of the Anaplasmataceae-positive samples from equids could be confirmed to species level by sequencing, probably due to low levels of bacteremia that prevented the generation of sufficiently amplified fragments. Nevertheless, the molecular infection frequency (50%) observed in this study exceeds that reported in other Latin American countries, such as Guatemala (13%), Chile (16.6%), and Costa Rica (14.6%) [17,58,59]. This finding contrasts with studies in Brazil [60] and Colombia [61] in which no infections were identified. There may be various reasons for these differences, including variations in study design, the number of animals analyzed, the epidemiological context and the diagnostic techniques and protocols employed.

The prevalence of Anaplasmataceae infection observed in the host species was high; nevertheless, risk factor analysis confirmed that the frequency of Anaplasmataceae in the animals tested was species-related, with a significantly higher prevalence found in cattle than in horses. This discrepancy may be indicative of vector host preferences and/or greater exposure of cattle to infected ticks, likely due to differences in grazing behavior and habitat use [54,62]. There may also be a direct correlation between the prevalence of tick infestation on farms and the observed infection risk in analyzed hosts. Specifically, the risk of Anaplasmataceae infection in individuals on farms where at least 78% of hosts were tick-infested was found to be 9.1 times higher compared to farms with a lower percentage of host infestation. This finding aligns with previous studies suggesting that high tick infestation increases the risk of Anaplasmataceae infection [24,49,55,63]. Indeed, the presence of persistently infected animals and the co-circulation of competent tick vectors on farms sustain ongoing Anaplasmataceae infection cycles [49,64]. These factors highlight the importance of effective management practices [24,49]. Measures that have been shown to reduce the tick burden and minimize the risk of Anaplasmataceae transmission include the strategic, selective use of chemical or biological acaricides to prevent resistance, as well as vaccinations against ticks, appropriate habitat and grazing management, and the use of tick-resistant breeds. These measures are particularly useful in endemic tropical areas where complete tick eradication is rarely feasible [65,66,67].

Several Anaplasmataceae species were identified in ticks and their hosts. While *Ehrlichia* sp., *E. ruminantium*, *E. muris*, and *Anaplasma* sp. (related to *A. platys*-like) were detected exclusively in ticks, *A. marginale* was found only in cattle, but not in the ticks infesting them. This is consistent with reports indicating that bovine ehrlichiosis is less prevalent than anaplasmosis [68]. Interestingly, none of the Anaplasmataceae species detected in cattle matched those identified in their associated tick pools. This discrepancy suggests a complex dynamic involving the acquisition of pathogens by ticks, influenced by several factors. These may include prior infections in hosts caused by previous tick bites, limited pathogen acquisition influenced by low rickettsemia levels, antigenic shifts or genetic variability in the pathogen during prolonged infections, or host immune responses that hinder pathogen uptake and reduce vector competence, among others [69,70,71,72,73].

The detection of *A. marginale* in cattle is consistent with reports from Brazil, Ecuador, and Colombia confirming its endemic circulation in tropical regions [16,19,55,63]. Its high and persistent prevalence in Colombia is largely driven by seasonal reinfections by infected ticks [49,54]. Phylogenetic analysis revealed genetically diverse *A. marginale* strains, suggesting the co-circulation of different genotypes. As observed in previous studies using different molecular markers, this genetic variability may be indicative of regional adaptation and varying pathogenicity [52,63,74,75].

The *Anaplasma* sp. sequence detected in *R. microplus* was closely related to *A. platys*-*like* strains that have previously been reported in ruminants in Egypt, Tunisia, Algeria, Senegal [42,76,77,78] and Italy [79]. *Anaplasma platys*, on the other hand, is primarily associated with *R. sanguineus* and is considered a canine pathogen [15], although infections have also been reported in humans [80,81,82] and in various tick species in Colombia [26,27,40,83,84]. There is documented evidence of *A. platys*-like organisms infecting cattle in Algeria and Brazil [42,85]. The pathogenicity of *A. platys*-like strains is not fully understood, but the close genetic relationship with *A. platys*, a known zoonotic species, and their wide host and vector range, suggest that they could have an epidemiologically relevant and geographically widespread distribution, which may include circulation in Colombia.

The *E. muris* isolate in the present study clustered with other strains previously identified in *Ixodes* ticks from the USA and rodents from Japan [86,87], showing low genetic variability and high bootstrap support (>80%). Its close relationship with the North American strain suggests that it could be classified as *E. muris* subsp. *Eauclairensis* [88]. To the best of our knowledge, this is the first report of an *E. muris*-like agent in Colombian ticks. However, as this identification is based on partial gene sequencing, further studies are necessary to confirm its presence and transmission dynamics.

We also report the first detection of *E. ruminantium* in Colombia. The sequence was strongly clustered (100% bootstrap) within the African clade [89,90,91] and showed low genetic variability. This pathogen, which is predominantly reported in Africa and the Caribbean, causes heartwater in ruminants. It is mainly transmitted by *Amblyomma* spp., although transmission by *Rhipicephalus* spp. has also been documented [67,92,93,94]. The detection of *E. ruminantium* in Colombia raises concerns about its potential establishment in the region and underscores its relevance as an agent of bovine ehrlichiosis. This finding is consistent with reports from Brazil [95,96], Canada [97] and also Africa [98].

In addition, we identified three *Ehrlichia* sp. that clustered with isolates related to *Ca*. *E. rustica* as well as species closely related to *E. canis* and *E. minasensis,* but distinct from the *E. minasensis* previously reported in *Hyalomma* and *Rhipicephalus* spp. from various regions of Africa and French Polynesia [90,99]. These sequences were identified in *R. microplus*, shared only 97% identity with previously reported strains [40] and exhibited moderate genetic divergence (0.02), which suggests the circulation of unclassified *Ehrlichia* agents in the Colombian Caribbean. Further molecular and phylogenetic studies are needed to validate this hypothesis.

The following limitations of the study should be acknowledged. First, the relatively small number of hosts analyzed, the limited set of sequenced samples selected from Anaplasmataceae-positive host–tick pairs, and reliance on a single genetic marker for species identification may have restricted the detection of additional genotypes or potential host–vector associations. These factors should be considered when interpreting the results. Second, the absence of serological testing limited the assessment of past exposure. Nevertheless, despite these constraints, the study provides novel molecular data on the circulation of Anaplasmataceae in the Colombian Caribbean and contributes valuable baseline data for forthcoming ecoepidemiological investigations. Future studies that integrate molecular and serological tools, analyze a larger number of samples, and include additional genetic markers to improve species resolution will be essential to clarify transmission dynamics and host–vector–pathogen interactions in the region.

## 5. Conclusions

This study enhances our knowledge and understanding of the prevalence and molecular characteristics of Anaplasmataceae in ticks and their host species in northern Colombia. It reveals high infection rates in vectors and host species, as well as circulation of both recognized and potentially novel Anaplasmataceae species. The detection of *Anaplasma* sp. closely related to *A. platys*-like, *E. muris*, *E. ruminantium*, and different *Ehrlichia* spp. in ticks highlights the complexity of the transmission dynamics and the possible zoonotic implications. This study represents the first regional investigation of the pathogen–vector–host interface, and provides a baseline for future studies with broader sampling and improved molecular characterization. Our findings underscore the need for expanded molecular surveillance and the inclusion of diverse tick and host species in diagnostic and control strategies, in accordance with the One Health framework.

## Figures and Tables

**Table 1:** Frequency of Anaplasmataceae infection in ticks and host animals in the Department of Atlántico, Colombia.

Ticks and Host Infected		n (%) (Developmental Stages)	No. Pools (%)	Positive (%)	CI_95%_	*p*-Value
**Tick species**	Rhipicephalus sanguineus * (cattle)	8 (0.7) (M:6, L:2)	2 (1.3)	0/2 (0.0)	0.0–0.0	<0.01
	Rhipicephalus microplus *	497 (43.0) (M:153, F:299, N:45)	82 (51.3)	15/82 (18.3)	10.0–27.0	
	Dermacentor nitens **	643 (55.6) (M:302, F:150, N:136, L:55)	70 (44.0)	0/70 (0.0)	0.0–0.0	
	Amblyomma patinoi *	8 (0.7) (M:1, F:7)	5 (3.1)	0/5 (0.0)	0.0–0.0	
	**Total**	**1156 (100)**	**159 (100)**	**15/159 (9.4)**	**5.0–14.0**	
**Host species**	Cattle	56 (76.7)	-	40 (71.4)	59.0–84.0	0.02
	Horse	14 (19.2)	-	7 (50.0)	15.0–67.0	
	Mule	3 (4.1)	-	0 (0.0)	0.0–0-0	
	**Total**	**73 (100)**	**-**	**47 (64.4)**	**53.0–76.0**	

M: male; F: female; N: nymph; L: larva. * Collected exclusively from cattle; ** Collected exclusively from horses and mules.

**Table 2:** Distribution of explanatory variables associated with Anaplasmataceae infection in sampled host species in the Department of Atlántico, Colombia.

Variable	Categories	Positive/Total	%	*p*
**Host species**	Cattle	40/56	71.4	0.025
	Equids	7/17	41.2	
**Age**	Juvenile (<3 years)	19/31	61.3	0.409
	Adult (≥3 years)	28/42	66.7	
**Sex**	Male	10/19	52.6	0.167
	Female	37/54	68.5	
**Location**	Metropolitan	13/18	72.2	0.014
	Eastern	8/17	47.1	
	South	6/7	85.7	
	Central	20/27	74.1	
	Coast	0/4	0.0	
**Infested animals on the farm (%)**	≥78%	28/35	80.0	0.017
	50–78%	12/21	57.1	
	<50%	7/17	41.2	
**Management system**	Extensive	11/14	78.6	0.185
	Intensive	25/37	67.6	
	Semiextensive	11/22	50.0	
**Tick control measures**	Pour on	2/5	40.0	0.238
	Pour on and by injection	45/68	66.2	
**Grazing rotation**	Yes	38/64	59.4	0.014
	No	9/9	100	
**Contact with wildlife**	Yes	27/46	58.7	0.142
	No	20/27	74.1	
**Type of feed**	Grass and forage	13/23	56.5	0.244
	Grass and concentrate	34/50	68.0	

**Table 3:** Multiple logistic regression analysis of risk factors associated with Anaplasmataceae infection in sampled animals in the Department of Atlántico, Colombia.

Variable	Categories	*p*-Value	OR (95% CI)
**Infested animals on the farm (%)**	≥78% 50–78% <50%	0.002 0.160 ^a^	9.1 (2.2–37.6) 2.7 (0.7–10.8) ^a^
**Host species**	Cattle Equids	0.008 ^a^	5.7 (1.6–20.9) ^a^

^a^ Reference Category.

## Data Availability

The data that supports the findings of this study are available from the authors upon reasonable request.
